# Stuck in Traffic—Myosin Motors Ease Gridlock in the Loop

**DOI:** 10.1093/function/zqaf008

**Published:** 2025-02-21

**Authors:** Joshua N Curry, James A McCormick

**Affiliations:** Division of Nephrology and Hypertension, Oregon Health and Science University, Portland, OR 97239, USA; Division of Nephrology and Hypertension, Oregon Health and Science University, Portland, OR 97239, USA

**A Perspective on “Thick Ascending Limb Specific Inactivation of Myh9 and Myh10 Myosin Motors Results in Progressive Kidney Disease and Drives Sex-specific Cellular Adaptation in the Distal Nephron and Collecting Duct”**

Kidneys regulate extracellular volume, salt, and water homeostasis through the complex interdependence of hemodynamics, tissue architecture, and the spatial distribution of tubule segments with distinct transport characteristics. The thick ascending limb (TAL) of the loop of Henle resides at the center of these processes, coordinating the dilution of tubular fluid and generation of a corticomedullary interstitial osmotic gradient. These functions, powered by apical sodium transport via the sodium-potassium-2 chloride cotransporter (NKCC2), are essential for urinary dilution and concentration, respectively.

At the cellular level, ion transport in the TAL is highly dynamic and involves the constant trafficking of proteins to and from the plasma membrane.^1^ Membrane trafficking is mediated in part by a family of proteins called myosins, molecular motors capable of binding to and moving along actin filaments using the hydrolysis of ATP.^.2^ Nonmuscle myosin II (NMII) is a ubiquitous class of myosins with key roles in epithelial cell function, including clathrin-dependent receptor-mediated endocytosis.^3^ While there are 3 known isoforms of NMII, in the renal epithelium, only NMII-A, encoded by the heavy chain gene *MYH9*, and NMII-B, encoded by the heavy chain gene *MYH10*, are expressed.^3^

In humans, *MYH9* mutations cause a spectrum of autosomal dominant hematologic disorders characterized by thrombocytopenia, giant platelets, and NMII protein aggregates within leukocytes, with variable renal involvement.^4^ It has been hypothesized that the severity of *MYH9-*related disease is determined by the propensity of the mutant protein to cause abnormal protein aggregates.^5^ Due to the redundant functions of NMII-A and NMII-B and the dominant negative effects of *MYH9* mutations on NMII function, Otterpohl et al. previously generated mice with conditional *Myh9*/*Myh10* knockout along the entire renal tubule to investigate the role of NMII proteins in the regulation of renal transport.^6^ Surprisingly, the mice exhibited progressive renal failure, while ion transport defects were mild or absent. The exception to this was the gradual loss of NKCC2 protein, associated with accumulation of uromodulin (UMOD) in TAL endoplasmic reticulum (ER).^6^ UMOD is a multifunctional glycosylphosphatidylinositol-anchored protein that is exclusively expressed in the TAL and the early segment of the distal convoluted tubule (DCT1).^7^

In this issue of *FUNCTION*, Otterpohl and colleagues^8^ report progressive renal failure due to tubulointerstitial fibrosis, immune cell infiltration, and tubular injury in mice with conditional deletion of *Myh9*/*Myh10* under control of the *Umod* promoter (*Myh9*/*Myh10* TAL-cKO). These findings recapitulate their earlier work, demonstrating accumulation of UMOD in TAL ER accompanied by calreticulin, a marker of ER stress, and upregulation of unfolded protein response proteins. Importantly, this model may be expected to induce genetic recombination in DCT1 given the expression of UMOD in this segment. However, Myh9 protein expression was absent in control DCT1 segments, while the Myh10 protein was preserved in DCT1 of *Myh9*/*Myh10* TAL-cKO animals. Indeed, the predominance of inflammation in the medulla and corticomedullary regions, along with the lack of effect on NCC expression, suggests that the major phenotype in this model originates from TAL dysfunction. While it is tempting to speculate that protein aggregation within TAL contributes to renal failure in patients with *MYH9*-related disease, most kidney biopsies from affected patients show glomerular pathology rather than tubulointerstitial disease,^4^ and it is unknown whether aggregation of Myh9 in TAL is a common feature of the disorder.

Otterpohl et al.^8^ highlight several additional findings, including the relatively rapid progression of renal failure and hypernatremia in female compared with male *Myh9*/*Myh10* TAL-cKO mice. Females exhibited earlier onset loss of NKCC2; whether this was due to more rapid protein aggregation in TAL remains untested, but it is established that female mice typically have higher expression of distal transport proteins compared with males.^9^ Alternatively, the differential injury between sexes could be explained by differences in inflammatory response or nephron adaptation. Despite the dramatic loss of NKCC2, *Myh9*/*Myh10* TAL-cKO mice did not exhibit the sodium wasting or hypokalemia typical of Bartter syndrome, caused by loss of function of NKCC2. Instead, they developed severe hypernatremia, which they could not compensate for with higher fluid intake. Given no significant changes in copeptin levels compared with control animals, the primary mechanism was likely vasopressin resistance, though this was not directly tested. While male *Myh9*/*Myh10* TAL-cKO developed polyuria and polydipsia concurrently with hypernatremia, females exhibited an earlier onset of hypernatremia in the absence of these findings. It should be noted that the animals also exhibited hypercalcemia relative to controls, and the study is limited by the lack of quantitative measurement of aquaporin-2. Ultimately, it is difficult to come to a clear conclusion regarding mechanisms of hypernatremia, perhaps due to confounding by these issues along with the severe reduction in renal function and moribund state of the animals at the time of collection.

An intriguing finding of this study is the identification of nephron adaptation in KO mice involving atypical expression of the γ subunit of the epithelial Na^+^ channel (γENaC) in the inner medulla. Interestingly, culture of the mouse inner medullary collecting duct cell line, IMCD3, demonstrated that culture under hypertonic conditions led to inhibition of γENaC mRNA expression. The authors speculate that loss of NKCC2 disrupts the corticomedullary interstitial gradient in *Myh9*/*Myh10* TAL-cKO mice, resulting in relative hypotonicity of the inner medulla and disinhibition of γENaC expression. Nephron remodeling is a well-recognized mechanism of diuretic resistance,^10^ and the authors propose a novel phenomenon by which NKCC2 dysfunction drives remodeling of sodium transport in the inner medulla that has potential relevance to treatment chronically with loop diuretics. Future work is warranted to assess the generalizability of this process and to study the role, if any, that neurohormonal activation plays in inner medulla remodeling.

Importantly, the phenotype of TAL-specific *Myh9*/*Myh10* kO mice shares striking similarity to patients with autosomal dominant tubulointerstitial kidney disease associated with rare mutations in the *UMOD* gene (ADTKD-*UMOD*). These patients develop progressive renal failure with tubulointerstitial inflammation, tubular dilatation, and mild urinary concentration defects due to accumulation of mutant UMOD protein in TAL ER.^7^ ADTKD-*UMOD* is just one of many examples of human proteinopathies for which novel therapies are sorely needed. Just as research on pathogenic protein folding and trafficking in cystic fibrosis has transformed modern treatment, advancing our understanding of these processes in renal proteinopathies could pave the way for novel therapeutic strategies.
